# Developing and maintaining the resilience of interdisciplinary cancer care teams: an interventional study

**DOI:** 10.1186/s12913-020-05882-3

**Published:** 2020-11-12

**Authors:** Carl-Ardy Dubois, Roxane Borgès Da Silva, Mélanie Lavoie-Tremblay, Bernard Lespérance, Kathleen Bentein, Alain Marchand, Sara Soldera, Christine Maheu, Sébastien Grenier, Marie-Andrée Fortin

**Affiliations:** 1 Département de gestion, d’évaluation et de politique de santé, École de santé publique de l’Université de Montréal, Montreal, Canada; 2 Centre de recherche en santé publique, CIUSSS du Centre-Sud-de-l’Île-de-Montréal et Université de Montréal, Montreal, Canada; 3 Ingram School of Nursing, McGill University, Montreal, Canada; 4 École des sciences de la gestion, Département d’organisation et ressources humaines, Université du Québec à Montréal, Montreal, Canada; 5 École de relations industrielles, Université de Montréal, Montreal, Canada; 6 Centre hospitalier Charles-Le Moyne, CISSS de la Montérégie-Centre, Longueuil, Canada; 7 Département de Psychologie, Université de Montréal, Montreal, Canada; 8 Centre intégré de cancérologie de Laval, CISSS de Laval, Laval, Canada

**Keywords:** Multidimensional intervention, Co-construction, Resilience, Occupational mental health, Oncology, Multidisciplinary team, Implementation analysis, Effects analysis, Cost-benefit analysis, Longitudinal design

## Abstract

**Background:**

Providing care to cancer patients is associated with a substantial psychological and emotional load on oncology workers. The purpose of this project is to co-construct, implement and assess multidimensional intervention continuums that contribute to developing the resilience of interdisciplinary cancer care teams and thereby reduce the burden associated with mental health problems. The project is based on resources theories and theories of empowerment.

**Methods:**

The study will involve cancer care teams at four institutions and will use a mixed-model design. It will be organized into three components:

***(1) Intervention development****.* Rather than impose a single way of doing things, the project will take a participatory approach involving a variety of mechanisms (workshops, discussion forums, surveys, observations) to develop interventions that take into account the specific contexts of each of the four participating institutions.

***(2) Intervention implementation and assessment****.* The purpose of this component is to implement the four interventions developed in the preceding component, assess their effects and whether they are cost effective. A longitudinal quasi-experimental design will be used. Intervention monitoring will extend over 12 months. The effects will be assessed by means of generalized estimating equation regressions. A cost-benefit analysis will be performed to assess the cost-effectiveness of the interventions, taking an institutional perspective (costs and benefits associated with the intervention).

***(3) Analysis of co-construction and implementation process****.* The purpose of this component is to (1) describe and assess the approaches used to engage stakeholders in the co-construction and implementation process; (2) identify the factors that have fostered or impeded the co-construction, implementation and long-term sustainability of the interventions. The proposed design is a longitudinal multiple case study.

**Discussion:**

In the four participating institutions, the project will provide an opportunity to develop new abilities that will strengthen team resilience and create more suitable work environments. Beyond these institutions, the project will generate a variety of resources (e.g.: work situation analysis tools; method of operationalizing the intervention co-development process; communications tools; assessment tools) that other oncology teams will be able to adapt and deploy elsewhere.

## Background

### Problem statement

Over the last decade, the well-being of cancer care workers, their resilience and their ability to deal with the hazards associated with complex care settings in this sector have emerged as major challenges for health care systems [1–3]. Cancer care workers take a heavy toll in terms of work-related mental health problems: stress and anxiety, fatigue, sense of ineffectiveness, burnout, depression [4, 5]. The significance of these problems is related not only to their potential impact on the quality of care, but also to the threats they pose to the viability of the workforce in this field. While the demand for cancer care treatment continues to rise, these problems are associated with an array of consequences (absenteeism, staff turnover, loss of productivity) that tend to shrink the pool of available workers [6, 7]. To be able to maintain a balance between the growing demand for increasingly complex oncology care and the supply of qualified human resources, decision makers must address the dual challenge of optimizing the health of their workers and strengthening the resilience of care teams.

### General research objective

The purpose of this project is to co-construct, implement and assess multidimensional intervention continuums that contribute to developing the resilience of interdisciplinary cancer care teams and thereby reduce the burden associated with mental health problems.

### Literature review

Recent studies show that the threats to the well-being of cancer care workers depend on interactions among a number of factors situated on three dimensions: (1) the nature of care and the associated emotional and psychological demands (e.g.: expectations of patients/families, complexity of treatments, possibility of death, feeling of helplessness, ethical dilemmas); (2) individual factors (e.g.: training, experience, work-life balance); (3) organizational conditions (e.g.: support of colleagues and immediate superior, workload, work organization, safety) [8–11]. These three dimensions refer to various situations that can be anticipated and averted in some cases, controlled in others, by strengthening team resilience [12]. Teams play a major role in complex settings such as cancer care where work demands often exceed individuals’ abilities and can only be met through interdependence-individuals working together. Organizations must rely on the collective abilities and resilience of their teams to deal with the variability of daily activities, the difficulty of the associated tasks, and the complexity of demands, often within a context of limited resources [13, 14].

Team resilience is not a new concept, but research work on operationalizing it in the workplace is still in the early stages of its development. While resilience is regarded as the outcome of interactions among an array of protective factors (psychological, social, organizational), interventions aimed at improving well-being do not always reflect this complexity. These interventions are often one-dimensional, targeting isolated factors (e.g.: interventions limited to primary prevention activities like training; interventions focused on individuals, ignoring organizational aspects and team dynamics) [15–17]. A systematic review, by Leppin et al. (2014), notes the lack of an integrating theoretical framework to guide the development of these interventions [18].

Despite a wide range of perspectives, conceptual and empirical research results on team resilience agree on two main aspects:

- The first is that team resilience is influenced by factors that operate at three levels: individual, team and organization. Team resilience reflects not only the characteristics of its individual members, but also the team’s distinctive qualities that emerge from interactions and transactions between members and that can take a variety of forms in various organizational settings [19, 20].
- The second aspect is that interventions to strengthen team resilience must be understood as a dynamic process of developing abilities that provide the team with the means to rise to challenges in a resilient fashion. These abilities have been grouped together into three categories: **(1) ability to monitor and anticipate hazardous situations and to prepare for them** by building a pool of available resources (personal, social, organizational); **(2) ability to respond to difficult situations** when they arise by tapping into the pool of resources; **(3) ability to learn from difficult situations** in order to generate new resources [12, 21].

This research suggests that multidimensional interventions, combining a variety of activities conducted at several levels, covering a continuum of abilities, and focused on the needs of stakeholders, constitute more effective options for building and maintaining team resilience. These studies also indicate, however, that the optimal combinations of activities are yet to be identified, owing to a small base of research with numerous limitations (static methods based on descriptive cross-sectional designs that clash with the dynamic nature of resilience; small sample sizes; short-term interventions and follow-up; qualitative assessments limited to the processes, without any analysis of effects) [4, 8, 16, 22].

### Theoretical framework

The project will be based on a theoretical body of work incorporating two perspectives: resources theories and theories of empowerment.

***Resources theories*** (demands-resources model, conservation of resources, job decision latitude) focus on the complexity of the determinants associated with mental health problems at work and the processes for developing resilience [23–25]. From this perspective, resilience depends on various personal (e.g.: stress management skills), social (e.g.: social network) and organizational (e.g.: support from supervisor) resources that teams can mobilize in hazardous situations. These resources can play a role as a buffer and provide protection against excessive demands or hazardous situations [26]. One characteristic of resilient teams is their ability to build a capacity pool and to tap into it when facing adverse situations [27, 28]. In situations where a loss of resources is inevitable, resilient teams stand out by their ability to control the management of these losses (redefine objectives, reallocate remaining resources to priority activities, review strategies and means available) and build new resources and capacity [29]. These theories will be useful, in this project, to help guide the construction of interventions in support of the development of various types of resources (personal, social, organizational) and to ensure balance with the demands teams must deal with.

***Theories of empowerment*** focus on the roles of the players involved in resilience development processes [30–32]. Contrary to top-down paternalist approaches, theories of empowerment take into account abilities that workers themselves can develop in conjunction with their employers to create more suitable work environments [33]. What make these theories particularly relevant is their holistic viewpoint that encompasses the ecology of the interventions, that is, the many factors that influence the problems and various perspectives of stakeholders [34]. They thus provide inspiration for an interactive approach that relies on multiple social and organizational mechanisms to foster intense interaction between the groups involved in the co-construction of the interventions [35].

## Methods

This study is intervention-oriented and will draw on scientific and experiential knowledge to co-construct, implement and assess interventions. It includes a variety of components based on those proposed by the National Occupational Research Agenda–Intervention Effectiveness Research: **(1)** Intervention development; **(2)** Intervention implementation and assessment (assessment of effects, economic assessment); **(3)** Implementation analysis [36]. The study will involve cancer care teams in four institutions (one CHU, two CIUSSS and one CISSS), which have played an active role in preparing this protocol.

### Component 1: intervention development

The factors that promote team resilience and the resources that protect against mental health problems in the workplace are fairly well known [19]. However, creating the conditions necessary for the emergence of these factors can equate to a wide range of activities and interventions. The optimal combinations of activities will ultimately depend on the specific characteristics of the teams involved, the characteristics of the individuals in the teams, the settings in which the teams operate and the challenges they face [13]. Rather than impose a universally applicable intervention, this project will opt for co-constructing an intervention that, within each institution, covers multiple dimensions while taking into account context specifics and the needs and preferences of stakeholders. Echoing the current state of knowledge about the development and maintenance of team resilience, each intervention will incorporate activities that contribute to the creation of the three aforementioned types of ability within teams: **(1) ability to monitor and anticipate** hazardous situations and prepare for them; **(2) ability to respond to** difficult, stressful situations when they arise; **(3) ability to learn from** difficult, stressful situations. The strategies and activities that can be deployed to develop these three forms of ability are summarized in the intervention framework set out below, which is adapted from the most recent developments on team resilience modelling [12, 37, 38] (Table 1).

**Table 1:** Intervention framework for the development of team resilience (adapted) [12, 37, 38]

Abilities	Strategies	Activities
**1. Ability to monitor and anticipate hazardous situations**	Identify hazards and anticipate challenges	• Main sources of stress team has had to deal with in the past • Main factors that can put stress on team in short and medium term • Types of situations team might have trouble dealing with • Means to be considered for dealing with current and anticipated sources of stress
	Assess team preparedness for hazardous situations	• Main points of team vulnerability (resources, expertise, support, collaboration) • Needs for dealing with current and anticipated challenges (training, coaching)
	Set up information and alert system	• Identify key indicators that can alert to potential problems (e.g., absenteeism, staff turnover, measurement of burnout) • Implement a regular system of data collection, processing and feedback on targeted indicators for teams
**2. Ability to respond to hazardous situations**	Empower teams and their members	• Workshops and simulation exercises for teams (problem, stress and demand management) • Workshops and simulation exercises for team leaders • Training and deployment of coaches (recruited from among teams, partner organizations, patient partners) • Build a resource and expertise bank (internal and external)
	Optimize team operating processes	• Accessibility of various tools for responding to targeted hazardous situations (e.g., protocols, guidelines) • Contingency plans/protocols for dealing with what are deemed top-priority situations • Clarification of responsibility for taking charge of hazardous situations • Familiarity with communication and coordination mechanisms and structures • Formal and informal discussion and interaction opportunities (team meetings, committees, ad hoc workshops, opportunities to socialize)
	Provide support for workers who are vulnerable or incapacitated	• Co-development of accommodation protocols (mechanisms, criteria, etc.) • Co-development of procedures for retention at work and return to work • Consolidation of support mechanisms during periods of disability
**3. Ability to learn from hazardous situations**	Conduct systematic analyses of adverse events	• Clarification of responsibilities with respect to review and analysis • Mechanisms of implementing review and analysis (development of analytical tools; team debriefing) • Process of identifying and implementing changes based on results of analysis

Based on the most recent research and the theoretical body of work underlying this project, the above intervention framework proposes an integrated range of activities that can be deployed to build team resilience. It does not prescribe a unique recipe that organizations must apply to the letter. Teams can instead use this framework to co-construct a multidimensional continuum of activities suited to their needs and their context. This continuum will, subsequently, be the object of a continuous assessment throughout the experimentation period to gauge its effectiveness and make adjustments to it if necessary.

Furthermore, while teams are targeted as the key units of intervention, the aforementioned activities form a continuum that covers three levels of ability development and takes into consideration the interdependence between the three levels: **(1) individuals** (e.g.: empowerment in the management of problems, demand and stressful situations); **(2) teams** (e.g.: analysis of sources of stress; team operating processes; debriefing after adverse events); **(3) organizations** (e.g.: retention at and return to work procedures; accommodation mechanisms).

The co-construction process will especially help in building the first type of ability defined in the intervention framework: *the ability to monitor and anticipate hazardous situations, but also to prepare an action plan.* The plan must set out how to respond to hazardous situations and develop the other two levels of ability. Co-construction will be deployed in each of the four institutions and will begin with a communications strategy that includes providing each team member with an information brochure, putting up posters at strategic locations and holding short information sessions. The process will then involve three steps:

1. ***Detailed analysis of work situations***: This analysis will lead to three types of activities:

- *A baseline survey* (**T0**). It will target all cancer care team workers in each institution and will cover the following variables, reflecting the theoretical frameworks used: (1) worker resources (personal resources measured by the work ability index [39]; social network [40]; organizational resources measured in terms of the support of supervisors and peers and decision latitude) [41]; (2) work-related demands (psychological and emotional demands [42] and work-family conflict) [43]; (3) effects of work environment (team resilience [37]; burnout [44]; perceived effectiveness of team) [45, 46]; (4) sociodemographic characteristics (age, sex, education, immigration status); (5) job attributes (job category, status, working hours) [47].
- *Collection of administrative data*. Collection will document the following for the last 12 months: labour participation of the various groups of workers (hours worked, days absent), workplace accidents, wage-loss benefits by causes. Relevant documentation will also be collected: organization charts, make-up and deployment of teams (e.g.: by work shift, by activity or geographic area, immediate superior), job structure, activity planning, minutes of meetings, etc.
- *Non-participant observations.* Based on a systematic chart, 14 h of observation will be carried out in each organization and will be planned so as to cover the different team practice settings. These observations will document physical work environments, interactions between team members, team structure and operating mechanisms, work rate, interruptions, tools and work processes.

This work situation analysis will provide outputs that will be used in the first co-construction workshop to identify problems that deserve priority attention.

(2) ***Preparation for co-construction***: This stage will involve the following activities: (a) selection of co-construction participants (a maximum of 15 participants will be recruited so as to include representatives of the various job groups and areas of activity, including doctors and representatives of human resources (HRD) and nursing; a patient partner will also be recruited to ensure that the patient’s perspective is represented in discussions); (b) preparation of documentation (summary of data from work situation analysis; (c) a facilitation team will be formed that will include a specialized facilitator, research team members and other specialized resources (e.g.: experts in ergonomics and organizational development) who can be drawn from resources already available in HR departments.
(3) ***Hold six co-construction workshops in each institution:*** The workshops will extend over 4 months. The first will take 2 h. The other five will be limited to 1 h, as agreed with the workplaces, for feasibility reasons:

- *Workshop 1. Define the problems to be solved.* This workshop will give participants an opportunity to think about their work environments, take stock of the current situation and identify the problems. It will be based on the results of the work situation analysis. In light of the aforementioned intervention framework, the workshop will be used to identify and anticipate situations that may be hazardous to teams and to define intervention priorities.

#### Deliverable: A well-defined set of two or three problems that will constitute the targeted priorities for the intervention

(b) *Workshop 2. Define the targeted objectives.* This workshop will focus on imagining the desired future and deciding on a roadmap for tackling current and anticipated challenges. Based on the intervention framework and taking into account the priorities defined in workshop 1, the purpose of this second workshop will be to define the immediate and ultimate objectives, with a view toward strengthening team resilience and mental health.

#### Deliverable: SMART objectives (specific, measurable, achievable, realistic, timely) with respect to each targeted priority

(c) *Workshops 3 and 4. Determine what actions need to be taken to achieve the objectives*. In these workshops, a continuum of activities that address the targeted problems will be defined. The activities will be selected on the basis of the three types of abilities set out in the intervention framework and these four criteria: potential effectiveness; feasibility; acceptability and interest for workers; assessability.

#### Deliverable: an integrated set of strategies and activities for achieving the objectives

(d) *Workshops 5 and 6. Specify the resources to draw on and the critical implementation conditions.* These workshops will serve to specify the resources to be mobilized in connection with the activities and objectives and to identify the critical conditions for implementation. The expected outcome is a logical intervention model (Problem-Objectives-Strategies-Activities-Resources) combined with an action plan and a schedule.

#### Deliverables: an action plan and an implementation schedule

The workshop facilitation techniques will include brainstorming activities, focus group and plenary discussions, an iterative modelling process that will result in an action plan. To expand participation and gather comments on the results of each workshop, three additional strategies will be used: a virtual discussion forum open to all workers concerned; short focus group discussions (30 min) during lunch with team members; and specific consultations with key respondents.

### Component 2: intervention implementation and assessment

#### Objectives

While component 1 will identify the priority problems and develop an action plan for addressing them (first type of ability in intervention framework), component 2 will directly concern the second and third types of abilities: *ability to respond to hazardous situations* by implementing co-constructed interventions and *ability to learn from difficult situations that arise*.

Three objectives are targeted: (1) Implement the intervention continuums developed by and specific to each of the four institutions; (2) Assess the effects of the interventions on team resilience, well-being, perceived effectiveness and absenteeism; (3) Assess the cost-benefit ratio of the interventions.

## Methods

### Objective 1: implementation

The intervention will involve all the workers who are members of cancer care teams in the participating institutions, including doctors. Team members are divided up into various job categories, each one having its own specific characteristics. However, in this research project, the main focus is on teams and the interdependencies between their members. From this perspective, all job categories will be taken into consideration.

Following the workshops, 2 months will be devoted to preparing for the implementation. In addition to mobilizing resources, preparation will involve two main activities:

#### Dissemination of the action plan

A short information brochure will be put together to present the action plan developed in the workshops and be distributed to the workers concerned. Short meetings will also be organized with key stakeholders (e.g.: unions, departments affected) which will be asked to pass on the information.

#### Establishment of a project structure in each institution

This structure will consist of:

- *A steering committee*, which will have a strategic role. It will be made up of the local clinical researcher or department head, a cancer care coordinator, HR and nursing department representatives, an appointed local union rep, an academic researcher and representatives of any other relevant stakeholders.
- *A local implementation committee (project management)*, which will have an operational role. It will consist of a project manager (from the institution or recruited in conjunction with it), a cancer care coordinator, a worker representative and a researcher.
- *Employee-ambassadors*. Three to five ambassadors, from the various job categories, will be recruited in each institution, and their role will be to motivate their co-workers to take part in the activities.

Implementation will take 6 months and will be organized in accordance with the action plan and schedule developed in the workshops. Follow-up will extend over 12 months. The project manager will work closely with the cancer care coordinator, under the supervision of the department head, and will be responsible for overseeing the implementation of the activities set out in the action plan. The local implementation committee will meet once a month to ensure effective coordination of all activities. A statutory meeting of the steering committee will be held quarterly to assess progress and make whatever adjustments are required. Throughout implementation, employee-ambassadors, union representatives and managers involved will be supplied with appropriate materials regarding activities underway in order to inform the support they should provide.

### Objective 2: assessment of effects

#### Design and target population

A longitudinal quasi-experimental design will be used. The target population is all workers in cancer care teams in the participating institutions: 240 in the first one (integrated health and social services network) 300 at the second [university hospital], 125 and 30 respectively in the two others (integrated health and social services university networks). The exact number of workers who will be directly impacted by the interventions in each setting will be defined on the basis of the work situation analysis and the action plan developed in the workshops (component 1 of the project).

#### Sample

Workers from the four institutions will be asked to respond to a questionnaire. Even with a conservative response rate of 50% of people answering at all the measurement times, we have calculated that, on the basis of a significance level of 0.05, a power of 80% and an intra-subject correlation of 0.5, our study will be able to detect a standardized difference between two measurement times (d of Cohen) equivalent to 0.26 (institution 1), 0.24 (institution 2), 0.36 (institution 3) and 0.78 (institution 4) standard-deviation units. This corresponds to a low effect size, which is very good, for the first three institutions [48]. The assessment of effects will therefore focus primarily on these first three institutions. For the last one, we will limit ourselves to tests on small sample sizes (e.g.: Wilcoxon test).

#### Variables and instruments

The effects of the intervention will be assessed on the basis of **four dependent variables**: team resilience [37], perceived effectiveness of team [45, 46], well-being at work measured by burnout [44] and absenteeism. In addition to the intervention itself, **the independent variables** considered will be (1) worker resources (personal resources measured by work ability [39]; social network [40]; organizational resources measured in terms of the support of supervisors and peers and decision latitude;) [41] (2) work-related demands (psychological and emotional demands [42] and work-life conflict;) [43] (3) sociodemographic characteristics (age, sex, education, immigration status); (4) job characteristics (job category, status, working hours;) [47] (5) degree of implementation of intervention (level of effective implementation of action plan developed in workshops). To measure this last variable, we will use the method proposed by Steckler and Linnan [49]. It combines two elements: the administered intervention dose (the number of activities conducted in relation to those planned) and the dose actually received (worker participation in the activities). The exact details regarding how these two elements will be measured will be adjusted on the basis of the various action plans.

#### Data collection

The strategy will include:

- *A repeated questionnaire*: To be completed just before the start of the intervention (**T1**), 6 months after the start of the intervention (**T2**, all activities will have begun) and at 12 months (**T3**, full impact of intervention). The questionnaire will be the same one that was used for component 1 of the project (**T0**) and will cover all the aforementioned variables except absences.
- *Collection of administrative data* on hours worked and absences, covering 12 months before the intervention and up to 12 months after the start of the intervention.

#### Data analysis

The effects of the dependent variables (resilience, perceived effectiveness of team, burnout, absenteeism) will be assessed by means of generalized estimating equation regressions with a non-structured correlation matrix. This type of model is particularly robust and well suited to longitudinal data [50]. It will allow the various measurement times to be analysed simultaneously without imposing unrealistic constraints on the data. Regression models will be constructed for each site and each dependent variable, taking into account the intervention and the other independent variables mentioned above.

### Objective 3: economic assessment

#### Design

A cost-benefit assessment will be conducted, as it is particularly well suited for assessing the cost-effectiveness of an intervention [51]. The assessment will compare the costs and consequences of the intervention with the costs and consequences of doing nothing, based on the following time references: the 12 months preceding the start of the intervention and the 12 months following the implementation of all the activities provided for in the action plans. The analysis will take into account the trends observed during the year.

#### Variables

The assessment will be carried out from the standpoint of the institution, by sticking to monetizable costs and benefits only. The direct and indirect costs of the interventions will be identified, the value of them estimated, measured and aggregated to arrive at the total cost [52].

The direct costs will include the costs of the resources associated with developing the intervention: (1) human resources (project manager, workers taking part in workshops, facilitators); (2) physical resources (materials, equipment). The direct costs will also include the costs associated with implementing the intervention. They will vary from one setting to the next depending on the intervention chosen.

The indirect costs will essentially consist of the costs associated with replacing the team members who will be taking part in developing the intervention (workshops) or in action plan activities.

The consequences will be measured on the basis of three parameters: benefits paid for absences from work; costs of replacing employees absent from work; costs of recruiting and integrating replacements.

#### Data collection

Data on direct and indirect costs will be collected using a structured form that will be filled in by the respondents involved (e.g.: cancer care department, HRD). Data on consequences will be compiled from HRD administrative data.

#### Analysis

The consequences of the intervention will be compared with the previous year. The monetary valuation for these two periods will be done in terms of constant dollars, which will be sufficient to make the two close periods comparable without needing to apply a discount rate. The cost-effectiveness of the intervention will be calculated using the cost-benefit ratio.

### Component 3: analysis of co-construction and implementation process

#### Objectives

The purpose of this component is to (1) describe and assess the approaches used to engage stakeholders in the co-construction and implementation of the interventions; (2) identify the factors that have fostered or impeded the co-construction, implementation and long-term sustainability of the interventions.

#### Design

A longitudinal multiple case study is proposed. The cases will be the cancer care teams at each of the participating institutions.

#### Data collection

Case construction will essentially involve qualitative data collected from a variety of sources: workers in the various job categories, individuals involved in co-construction or implementation, managers (*n* = 12 for each case, to the point of data saturation). The collection methods will be interviews and compiling the relevant documentation. The interviews will be repeated, at two times: three and 12 months after the start of the intervention. The interview guide will cover numerous aspects: resources invested, actors mobilized, implementation methods, facilitating and constraining factors for individuals and teams.

#### Analysis

Once the transcriptions have been coded (Atlas.ti software), the data processing will involve narrative analysis [53] and constant comparative method [54] techniques. The initial aim of the analysis will be to describe the observations for each case. Comparative analysis will then be used to compare the cases and identify similarities and differences. The longitudinal analysis will use narrative investigation approaches to reconstitute the sequence of main events and the links between them [55].

## Discussion

A number of strategies have been used, both in the design of the project and in its rollout and implementation, to ensure its feasibility and long-term sustainability. In the participating institutions, the project has been placed at the top of management committees’ strategic agenda, with commitments to it having been made at the highest level of the cancer care, human resources and nursing departments and by chief executive officers. The project takes a participatory approach that engages all key stakeholders. This ensures that the targeted problems and objectives are consistent with organizational priorities and that the proposed interventions are feasible and broadly accepted. To optimize coordination of project implementation, we will build on the following structure: steering committee, local implementation committee and employee ambassadors. These committees are designed to ensure continuous liaison with permanent stakeholders, which will take over at the end of the project cycle. The make-up of these committees will be adapted on the basis of local characteristics. Implementing the action plans resulting from the workshops will depend in large part on contributions in kind by the institutions and will be consistent with the mission of the human resources departments. Our approach is to serve as a catalyst for the development and implementation of new interventions by providing the support required to strengthen resilience abilities. It will enable cost-effective interventions that maximize the use of internal resources and improve existing processes.

In addition to knowledge production, the project will have direct and indirect benefits for the teams and the institutions, both in the short and long term.

In the short term, the project is expected to have beneficial impacts at three main levels:

- *A reduction in the burden associated with mental health problems in interdisciplinary cancer care teams.* Four co-constructed interventions will be rolled out respectively in four institutions, with the objective of not only strengthening team resilience, but also of maintaining team mental health and optimizing work participation. Implementing these interventions will thereby help reduce both the monetary costs (absences from work, lost productivity) and the human burden (lower quality of life) associated with mental health problems.
- *A strengthening of the abilities of cancer care teams and their organizations*. The empowerment perspective that guides this project means that stakeholders develop the abilities required so that they themselves can create more suitable work environments. The project provides many opportunities to develop these abilities. The proposed co-construction approach gives organizational players a unique learning opportunity to draw up, experiment with and refine solutions to problems in their work environment. The intervention framework that provides guidance for intervention content will serve to build a continuum of three types of abilities that help maintain resilience.
- *Building a toolbox that could be used by all cancer care teams in Quebec and elsewhere to develop their resilience and revitalize their workplaces*. Beyond the participating institutions, the same approach could be adopted by other teams to develop innovative interventions based on reflective practices. The various outcomes of this study will constitute tools that can be adapted and deployed by other cancer care teams throughout Quebec: work situation analysis tools; methodology of operationalizing the co-construction approach; facilitation tools; communications tools; action plans; intervention implementation tools; assessment tools. The project will be carried out in four institutions whose workplace settings differ in a number of respects: institution’s mission and clienteles, team size, team composition, organization of cancer care services. This kind of variety necessarily increases the range of institutions that could take this project as a basis for designing their own intervention.

Over the longer term, this proposal lays the foundations for a more coordinated, more comprehensive, systemic approach toward occupational health interventions. It will thereby help foster the development of organizational practices and human resources policies that give greater weight to the multidimensional nature of occupational health issues, with respect to both their determinants and their consequences. The adoption and institutionalization of the proposed approach in a growing number of institutions and the long-term sustainability of the resulting interventions will help make lasting improvements in cancer care work environments, with the potential of ensuring greater viability of the workforce in this field. Beyond cancer care, the project will also serve as a springboard to help organizations develop better collaborative and interdisciplinary intervention practices. The lessons learned in cancer care could be extended to other areas facing similar challenges (e.g.: palliative care). Transferability to other areas will be facilitated in particular if human resources departments and nursing departments are involved.

As in other areas of care, patient well-being in cancer treatment is closely linked to the well-being of the professional staff. In addition to its impact on work environments, this project will establish a set of conditions (staff retention and stability, maintaining staff health, work attendance) that will improve the quality of care for cancer patients.

## Data Availability

Although data collected for this study will be stored for up to 7 years, the datasets generated and analyzed will not be available to people outside the research team, due to conditions of the ethical approvals. However, detailed information on the analyses conducted will remain available.

## Abbreviations

CHU: Centre hospitalier universitaire (university hospital center)
CIUSSS: Centre intégré universitaire de santé et de services sociaux (integrated university health and social services centre)
CISSS: Centre intégré de santé et de services sociaux (integrated health and social services centre)
HRD: Human resources department

## References

[1] Cañadas-De la Fuente GA, Gómez-Urquiza JL, Ortega-Campos EM, Cañadas GR, Albendín-García L, De La Fuente-Solana EI (2018). Prevalence of burnout syndrome in oncology nursing: a meta-analytic study. Psychooncology..

[2] Mukherjee S, Beresford B, Glaser A, Sloper P (2009). Burnout, psychiatric morbidity, and work-related sources of stress in paediatric oncology staff: a review of the literature. Psychooncology..

[3] Sehlen S, Vordermark D, Schäfer C, Herschbach P, Bayerl A, Pigorsch S, Rittweger J, Dormin C, Bölling T, Wypior HJ, Zehentmayr F, Schulze W, Geinitz H (2009). DEGRO quality of life work group. Job stress and job satisfaction of physicians, radiographers, nurses and physicists working in radiotherapy: a multicenter analysis by the DEGRO quality of life work group. Radiat Oncol.

[4] Kutluturkan S, Sozeri E, Uysal N, Bay F (2016). Resilience and burnout status among nurses working in oncology. Ann Gen Psychiatry.

[5] Medisauskaite A, Kamau C (2017). Prevalence of oncologists in distress: systematic review and meta-analysis. Psychooncology..

[6] Erikson C, Salsberg E, Forte G, Bruinooge S, Goldstein M (2007). Future supply and demand for oncologists: challenges to assuring access to oncology services. J Oncol Pract..

[7] Toh SG, Ang E, Devi MK (2012). Systematic review on the relationship between the nursing shortage and job satisfaction, stress and burnout levels among nurses in oncology/haematology settings. Int J Evid Based Healthc.

[8] Murali K, Makker V, Lynch J, Banerjee S (2018). From burnout to resilience: an update for oncologists. Am Soc Clin Oncol Educ Book.

[9] Le Blanc PM, Bakker AB, Peeters MC, van Heesch NCA, Schaufeli WB (2001). Emotional job demands and burnout among oncology care providers. Anxiety Stress Coping.

[10] Potter P, Deshields T, Divanbeigi J, Berger J, Cipriano D, Norris L, Olsen S (2010). Compassion fatigue and burnout: prevalence among oncology nurses. Clin J Oncol Nurs.

[11] Najjar N, Davis LW, Beck-Coon K, Doebbeling CC (2009). Compassion fatigue: a review of the research to date and relevance to cancer-care providers. J Health Psychol.

[12] Alliger GM, Cerasoli CP, Tannenbaum SI, Vessey WB (2015). Team resilience: how teams flourish under pressure. Organ Dyn.

[13] Gucciardi DF, Crane M, Ntoumanis N, Parker SK, Thøgersen-Ntoumani C, Ducker KJ, Peeling P, Chapman MT, Quested E, Temby P (2018). The emergence of team resilience: a multilevel conceptual model of facilitating factors. J Occup Organ Psychol.

[14] Amaral A, Fernandes G, Varajão J (2015). Identifying useful actions to improve team resilience in information systems projects. Procedia Comput Sci.

[15] Back AL, Steinhauser KE, Kamal AH, Jackson VA (2017). Why burnout is so hard to fix. J Oncol Pract.

[16] Panagioti M, Panagopoulou E, Bower P, Lewith G, Kontopantelis E, Chew-Graham C, Dawson S, van Marwijk H, Geraghty K, Esmail A (2017). Controlled interventions to reduce burnout in physicians: a systematic review and metaanalysis. JAMA Intern Med.

[17] Moore P, Rivera S, Bravo-Soto GA, Olivares C, Lawrie TA (2018). Communication skills training for healthcare professionals working with people who have cancer. Cochrane Database Syst Rev.

[18] Leppin AL, Bora PR, Tilburt JC, Gionfriddo MR, Zeballos-Palacios C, Dulohery MM, Sood A, Erwin PJ, Brito JP, Boehmer KR, Montori VM (2014). The efficacy of resiliency training programs: a systematic review and meta-analysis of randomised controlled trials. PLoS One.

[19] Bowers C, Kreutzer C, Cannon-Bowers J, Lamb J (2017). Team resilience as a second-order emergent state: a theoretical model and research directions. Front Psychol.

[20] Kennedy DM, Landon LB, Maynard MT (2016). Extending the conversation: employee resilience at the team level. Ind Organ Psychol.

[21] Lengnick-Hall CA, Beck TE, Lengnick-Hall ML (2011). Developing a capacity for organizational resilience through strategic human resources management. Hum Resour Manag Rev.

[22] Awa WJ, Plaumann M, Walter U (2010). Burnout prevention: a review of intervention programmes. Patient Educ Couns.

[23] Demerouti E, Bakker AB, Nachreiner F, Schaufeli WB (2001). The job demands-resources model of burnout. J Appl Psychol.

[24] Hobfoll SE (1989). Conservation of resources: a new attempt at conceptualizing stress. Am Psychol.

[25] Karasek RA (1979). Job demands, job decision latitude, and mental strain: implications for job redesign. Adm Sci Q.

[26] Hobfoll SE, Folkman S (2010). Conservation of resources theory: its implication for stress, health, and resilience. The Oxford handbook of stress, health, and coping.

[27] Sutcliffe KM, Vogus TJ, Cameron K, Dutton JE, Quinn RE (2003). Organizing for resilience. Positive organizational scholarship.

[28] Stoverink AC, Kirkman BL, Mistry S, Rosen B. Bouncing back together: toward a theoretical model of work team resilience. Acad Manage Rev. 2020;45(2):395–422.

[29] Vera M, Rodríguez-Sánchez A, Salanova M (2017). May the force be with you: looking for resources that build team resilience. J Workplace Behav Health.

[30] Spreitzer GM (1996). Social structural characteristics of psychological empowerment. Acad Manag J.

[31] Crawford Shearer NB (2009). Health empowerment theory as a guide for practice. Geriatr Nurs.

[32] Kanter RM (1993). Men and women of the corporation.

[33] Wallerstein N (1992). Powerlessness, empowerment, and health: implications for health promotion programs. Am J Health Promot.

[34] Rogers M, Riehl JP, Roy C (1980). A science of unitary man. Conceptual models for nursing practice 2.

[35] Dubois C-A, Contandriopoulos D (2010). La gestion des connaissances: un concept aux multiples facettes qui donne lieu à de multiples stratégies. Le Point en administration de la santé.

[36] Goldenhar LM, LaMontagne AD, Katz T, Heaney C, Landsbergis P (2001). The intervention research process in occupational safety and health: an overview from the National Occupational Research Agenda Intervention Effectiveness Research team. J Occup Environ Med.

[37] Van der Beek D, Schraagen JM (2015). ADAPTER: Analysing and developing adaptability and performance in teams to enhance resilience. Reliability Eng Syst Safety.

[38] Patriarca R, Di Gravio G, Costantino F, Falegnami A, Bilotta F (2018). An analytic framework to assess organizational resilience. Saf Health Work.

[39] Ilmarinen J (2007). The work ability index. Occup Med (Lond).

[40] Stevens N, Westerhof GJ (2006). Partners and others: social provisions and loneliness among married Dutch men and women in the second half of life. J Soc Pers Relat.

[41] Karasek R, Brisson C, Kawakami N, Houtman I, Bongers P, Amick B (1998). The job content questionnaire (JCQ): an instrument for internationally comparative assessments of psychosocial job characteristics. J Occup Health Psychol.

[42] Albrecht SL (2015). Challenge demands, hindrance demands, and psychological need satisfaction: their influence on employee engagement and emotional exhaustion. J Personnel Psychol.

[43] Netemeyer RG, Boles JS, McMurrian R (1996). Development and validation of work-family conflict and family-work conflict scales. J Appl Psychol..

[44] Maslach C, Jackson SE (1986). Maslach burnout inventory manual.

[45] Temkin-Greener H, Zheng N, Katz P, Zhao H, Mukamel DB (2009). Measuring work environment and performance in nursing homes. Med Care.

[46] Shortell SM, Marsteller JA, Lin M, Pearson ML, Wu SY, Mendel P, Cretin S, Rosen M (2004). The role of perceived team effectiveness in improving chronic illness care. Med Care.

[47] Vézina M, Cloutier E, Stock S, Lippel K, Fortin É, Delisle A (2011). Enquête québécoise sur des conditions de travail, d’emploi, et de santé et de sécurité du travail (EQCOTESST).

[48] Sawilowsky S (2009). New effect size rules of thumb. J Mod Appl Stat Methods.

[49] Linnan L, Steckler A (2002). Process evaluation for public health interventions and research.

[50] Hardin J, Hilbe J (2003). Generalized estimating equations.

[51] Drummond MF, Sculpher MJ, Claxton K, Stoddart GL, Torrance GW (2015). Methods for the economic evaluation of health care programmes.

[52] Canadian Agency for Drugs and Technologies in Health (2017). Guidelines for the economic evaluation of health technologies in Canada.

[53] Chase S (2005). Multiple lenses, approaches, voices.

[54] Boeije H (2002). A purposeful approach to the constant comparative method in the analysis of qualitative interviews. Qual Quant.

[55] Grossoehme D, Lipstein E (2016). Analyzing longitudinal qualitative data: the application of trajectory and recurrent cross-sectional approaches. BMC Res Notes.

